# Upregulation of hsa_circ_0007874 suppresses the progression of ovarian cancer by regulating the miR‐760/SOCS3 pathway

**DOI:** 10.1002/cam4.2866

**Published:** 2020-02-05

**Authors:** Li Li, Poling Yu, Ping Zhang, Huanmei Wu, Qizhen Chen, Shuangdi Li, Yanqiu Wang

**Affiliations:** ^1^ Department of Gynecology and Obstetrics Tongji Hospital Tongji University School of Medicine Shanghai China; ^2^ Department of Surgery School of Medicine National Yang‐Ming University Taipei Taiwan China; ^3^ Department of Reproductive Medicine Linyi People's Hospital Linyi City China; ^4^ Department of BioHealth Informatics School of Informatics and Computing Indiana University Purdue University Indianapolis Indianapolis IN USA; ^5^ Department of Gynecology Shanghai First maternity and infant Hospital Shanghai China; ^6^ Reproductive Medical Center Tongji Hospital Tongji University School of Medicine Shanghai China

**Keywords:** hsa_circ_0007874, miR‐760, ovarian cancer, SOCS3

## Abstract

Ovarian cancer (OVA) is a fatal and common malignancy in women worldwide. Circular RNAs (circRNAs) consist of a family of circular endogenous RNAs generated by selective splicing, and they are involved in many diseases. Previous studies reported that hsa_circ_0007874 is aberrantly expressed in cancer and functions in tumorigenesis. While the hsa_circ_0007874 role in OVA is unclear. Here, we detected the hsa_circ_0007874 expression in OVA cell lines using Rt‐qPCR. Hsa_circ_0007874 subcellular localization was confirmed by fluorescence in situ hybridization. The relationship between hsa_circ_0007874, microRNAs (miRNAs), and relative protein levels was assessed using the luciferase reporter assays. Results verified that hsa_circ_0007874 is downregulated in OVA cell lines. hsa_circ_0007874 overexpression decreased the OVA cell migration and proliferation in vitro and in vivo. Bioinformatics and luciferase reporter assays confirmed that miR‐760 and SOCS3 are the downstream targets of hsa_circ_0007874. Downregulation of SOCS3 or miR‐760 overexpression restored the migration and proliferation ability of SKOV3 or A2780 cells overexpressing hsa_circ_0007874. Downregulation of SOCS3 restored the proliferation and migration in miR‐760 knockdown SKOV3 and A2780 cells. In summary, the data suggest that hsa_circ_0007874 acts as a tumor suppressor by regulating the miR‐760/SOCS3 axis, highlighting its potential as an effective therapeutic target for OVA.

## Graphical abstract

Downregulation of SOCS3 or overexpression of miR‐760 restored the proliferation and migration ability of SKOV3 or A2780 cells overexpressing hsa_circ_0007874. Downregulation of SOCS3 restored the proliferation and migration in miR‐760 knockdown SKOV3 and A2780 cells.

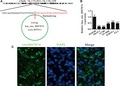

## 1 INTRODUCTION

Ovarian cancer (OVA) is a fatal and popular malignancy among women worldwide, accounting for 5%‐6% of cancer‐related deaths.^1^, ^2^ In 2017, it was estimated that 22 500 patients were diagnosed with OVA and 14 100 patients died from the disease in the United States.^3^, ^4^ Because of the vagueness of symptoms and the lack of early detection tests, 70%‐75% of OVA patients are at advanced stages when first diagnosed.^5^ The current standard therapeutic strategy for OVA is surgery combined with chemotherapy. However, approximately 80% of the patients develop resistance to treatment and metastasis.^6^, ^7^ This underscores the importance of early detection and the establishment of new therapeutic interventions for OVA.

Circular RNAs (circRNAs) are a new type of endogenous RNAs characterized by a covalently closed continuous loop. circRNAs function importantly in tumorigenesis in various malignancies, including clear cell renal cell carcinoma,^8^ gastric cancer,^9^ colorectal cancer,^10^ cervical cancer,^11^ and hepatocellular carcinoma.^12^ The expression of circRNA ABCB10 correlates with unfavorable survival and advanced clinicopathological features, and enhances cell proliferation by inhibiting apoptosis in epithelial OVA.^13^ A previous study validated that the circRNA hsa_circ_0007874 is aberrantly expressed in cancer and functions in tumorigenesis.^14^, ^15^, ^16^ However, the hsa_circ_0007874 role in OVA is unclear.

This study aimed to detect the hsa_circ_0007874 expression in OVA cell lines and explore the underlying mechanisms. Data indicated that hsa_circ_0007874 was downregulated in OVA, and hsa_circ_0007874 upregulation suppressed the cell proliferation and migration. These data supply a foundation for the novel therapeutic strategy development against OVA for clinical application.

## 2 MATERIALS AND METHODS

### 2.1 Animal ethics statement

BALB/c nude mice (n = 12) with 4 weeks old weighing 15‐20 g (SLARC) were utilized in the investigation. Ethics Committee in Shanghai Tongji Hospital, Tongji University, Shanghai, China approved all animal experiments.

### 2.2 Cell Culture

We cultured OVA cell lines (IGROV1, A2780, ES‐2, OV2008, and SKOV3) and the normal ovarian cell line ISOE80 in Dulbecco's Modified Eagle's Medium (DMEM; Gibco) supplied with fetal bovine serum (FBS; Gibco) of 10% and penicillin in a humidified incubator with 5% CO_2_ under 37°C.

### 2.3 Fluorescence in situ hybridization

A FITC‐labeled biotin‐labeled hsa_circ_0007874 probe (5′‐GGAAGGATTACATGACATCTGACCCAAAACAACCCCACTGAC‐3′‐biotin) was synthesized by Sangon Biotech. We counterstained nuclei with 4,6‐diamidino‐2‐phenylindole. We performed experiments following standard procedures (Genepharma).

### 2.4 Bioinformatics analysis

The interaction between SOCS3 3′‐untranslated region (3′‐UTR) and miR‐760 was predicted using the TargetScanHuman database (http://www.targetscan.org/vert_71/). The relationship between miR‐760 and hsa_circ_0007874 was predicted with (http://starbase.sysu.edu.cn/) database.

### 2.5 Migration assay

For cell migration analysis, we placed A2780 and SKOV3 cells into a Transwell upper chamber with density of 1 × 10^5^ cells (8 μm pore membrane; BD Biosciences) in 200 μL serum‐free medium. We added the complete medium (500 μL) to the bottom chamber. After 1 day of culture, we counted cell numbers in the bottom chamber after fixing with 4% paraformaldehyde and staining with crystal violet of 0.1%.

### 2.6 Metastasis assays and tumor xenograft formation

In total, we injected 2 × 10^7^ A2780 cells with or without hsa_circ_0007874 overexpression into nude mice right flanks.^17^ Tumor size (volume = 0.5 × length × width^2^) was measured using Vernier calipers for every 5 days for 1 month before mice were euthanized.

### 2.7 Dual luciferase reporter assay

We constructed the reporter plasmids by inserting the wild‐type or mutant circ‐0007874 or SOCS3 3'‐UTR sequence into a pGL3 vector (Promega). Lipofectamine 2000 was used to co‐transfect miR‐760 mimics and reporter plasmids into HEK239T cells. We assessed Firefly and Renilla luciferase activities using the Dual Luciferase Reporter Assay System (Promega) after culturing for 2 days.

### 2.8 Quantitative reverse transcription‐polymerase chain reaction (QRT‐PCR)

We extracted total RNA from cells using the TRIzol reagent (Invitrogen). We measured the RNA concentration using an ultraviolet spectrophotometer (Hitachi, Tokyo, Japan). We reverse transcribed RNA into cDNA through the Reverse Transcription Kit (TaKaRa Biotechnology Co., Ltd.). The thermocycling conditions were: 30 seconds at 95°C, 5s for 40 cycles at 95°C, and 35 seconds at 60°C. We calculated the relative expression via the 2^−ΔΔCt^ method. We used β‐actin as the internal reference. Experiments were repeated three times. The primer sequences used are: hsa_circ_0007874, forward 5′‐GCATCGGAAAGGGACATTTA‐3′, reverse 5′‐AGCTCTCAGACCCCACACAG‐3′; SOCS3, forward 5′‐GTCCCCCCAGAAGAGCCTATTA‐3′, reverse 5′‐TTGACGGTCTTCCGACAGAGAT‐3′; miR‐760, forward 5′‐CAGTCCCACAGCCTATCATCGATTGAAAAATCAAGGG‐3′, reverse 5′‐CCCTTGATTTTTCAATCGATGATAGGCTGTGGGACTG‐3′; GAPDH, forward, 5′‐TGTTCGTCATGGGTGTGAAC‐3′; reverse, 5′‐ATGGCATGGACTGTGGTCAT‐3′; U6, forward, 5′‐CTCGCTTCGGCAGCACA‐3′; reverse, 5′‐AACGCTTCACGAATTTGCGT‐3′.

### 2.9 Statistical analyses

GraphPad Prism (GraphPad) was employed for the analysis. Data were denoted by mean ± SEM *P‐*values ≤ .05 indicated significant differences.

## 3 RESULTS

### 3.1 hsa_circ_0007874 expression is decreased in OVA cell lines

**Figure 1:**
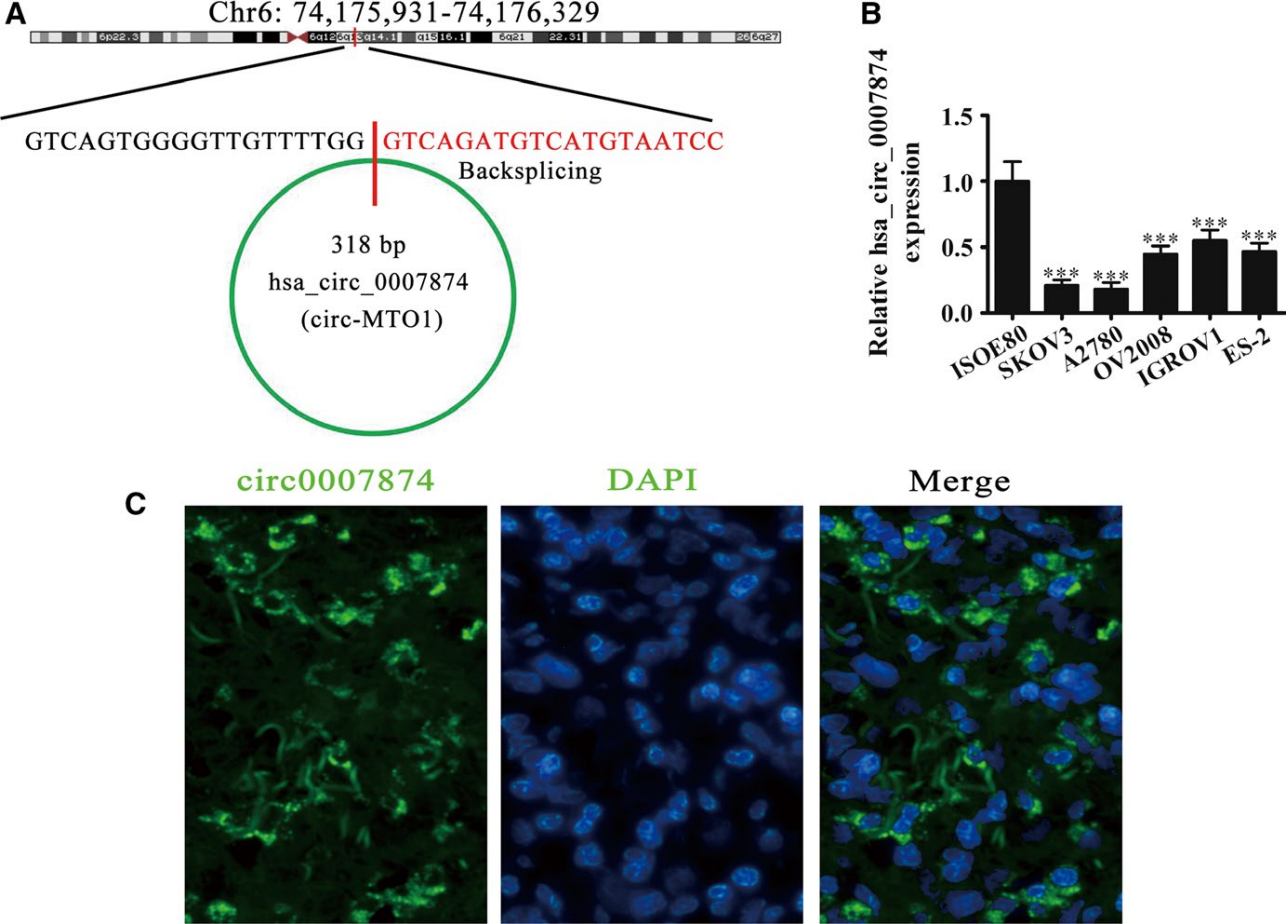
The expression and subcellular localization of hsa_circ_0007874 in OVA cell lines. A, Genomic loci of *MTO1* and hsa_circ_0007874. A red signal indicates back splicing. B, RT‐qPCR detection showing the expression of hsa_circ_0007874 in primary cultured normal ovarian epithelial cells and OVA cell lines. Data are presented as the mean ± SD. ***P < .001 vs normal. C, In situ hybridization was used to determine the subcellular localization of hsa_circ_0007874.

Previous studies showed that hsa_circ_0007874 has antitumor effects in lung adenocarcinoma^18^ and breast cancer.^19^ While the hsa_circ_0007874 role in OVA is unclear. Hsa_circ_0007874 is assembled with exons from the *MTO1* gene with a 318 bp splice length. Hsa_circ_0007874 is located at chr6:74175931‐74176329 (Figure 1A). Rt‐qPCR detection illustrated that the hsa_circ_0007874 expression was lower in OVA cell lines such like A2780, IGROV1, ES‐2, OV2008, and SKOV3 than in the normal ovarian cell line ISOE80 (Figure 1B). Because the hsa_circ_0007874 expression is particularly low in A2780 and SKOV3 cells, we used these cell lines for subsequent experiments. Fluorescence in situ hybridization assays illustrated that hsa_circ_0007874 predominately localized to the cytoplasm (Figure 1C).

### 3.2 Overexpression of hsa_circ_0007874 decreases the ova cell migration and proliferation ability

**Figure 2:**
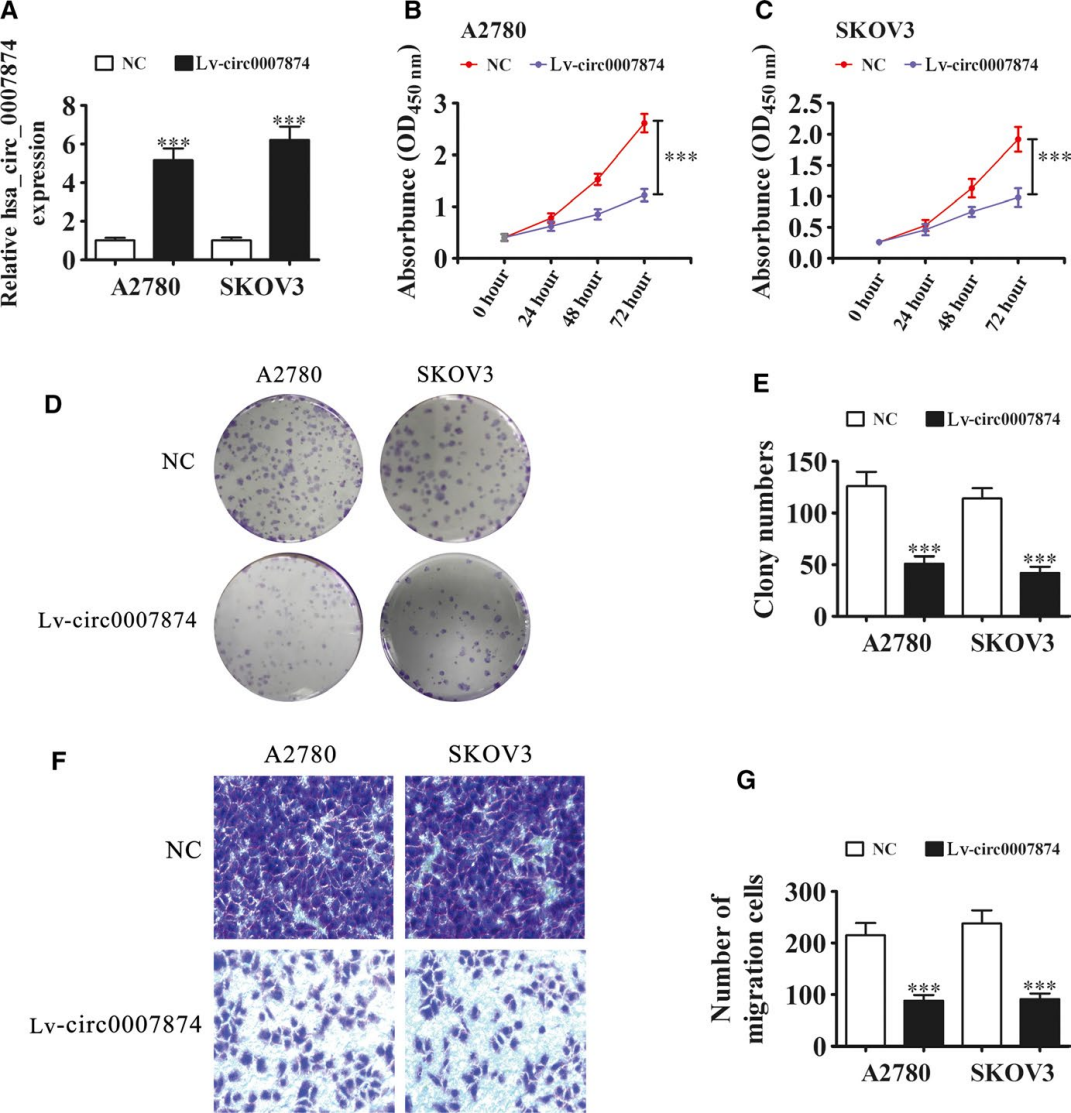
Overexpression of hsa_circ_0007874 decreased the OVA cell proliferation and migration ability. (A) qRT‐PCR detection showing the expression of hsa_circ_0007874 in A2780 and SKOV3 cells transfected with hsa_circ_0007874 overexpression vector (Lv‐circ0007874) or NC. Data are presented as the mean ± SD. ***P < .001 vs NC. (B‐E) Cell proliferation was analyzed by CCK‐8 assay (B and C) and colony formation (D and E). Data are presented as the mean ± SD. ***P < .001 vs NC. (F and G) Cell migration was assessed in both A2780 and SKOV3 cells using Transwell assays. Data are presented as the mean ± SD. ^***^ *P* < .001 vs NC

To detect the hsa_circ_0007874 role in the OVA progression, an hsa_circ_0007874 overexpression vector (Lv‐circ0007874) was constructed and transfected into A2780 and SKOV3 cells. Rt‐qPCR detection showed that the hsa_circ_0007874 expression was significantly higher in A2780 and SKOV3 cells transfected with Lv‐circ0007874 than in the negative control (NC) group (Figure 2A). CCK8 (Figure 2B,C) and clone formation (Figure 2D,E) assays verified that hsa_circ_0007874 upregulation suppressed the cell proliferation in A2780 and SKOV3 cells. Transwell migration assays demonstrated that the migratory capacity of A2780 and SKOV3 cells was diminished after hsa_circ_0007874 overexpression (Figure 2F,G).

### 3.3 SOCS3 and MIR‐760 are the downstream targets of hsa_circ_0007874

**Figure 3:**
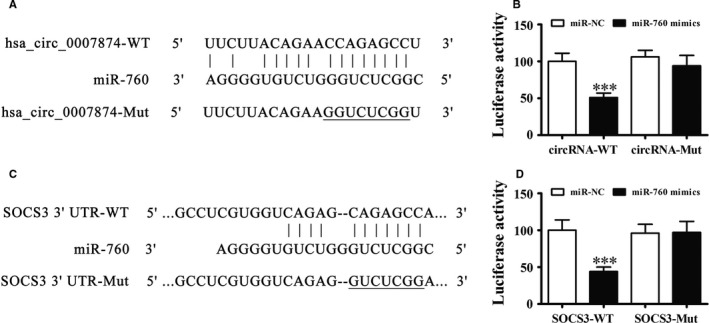
SOCS3 and miR‐760 are downstream targets of hsa_circ_0007874. A, The miR‐760 binding sites in hsa_circ_0007874 were predicted by bioinformatics analysis. Hsa_circ_0007874 mutated (Mut) was constructed. B, The relative luciferase activity was determined 48 h after transfection with miR‐760 mimic/normal control (NC) or with the hsa_circ_0007874 wildtype/Mut in HEK293T cells. Data are expressed as the mean ± SD. ^***^ *P* < .001. C, The predicted miR‐760 binding sites with the SOCS3 3'‐UTR. The 3'‐UTR‐SOCS3 mutated version is also provided. D, Relative luciferase activity was determined 48 h after transfection with miR‐760 mimic/normal control or with the 3'‐UTR‐SOCS3 wildtype/Mut in HEK293T cells. Data are expressed as the mean ± SD. ^***^ *P* < .001

Increasing evidence indicates that circRNAs are preferentially located and function in the cytoplasm, and they function as microRNA (miRNA) sponges to affect translation or bind directly to proteins regulating the protein function and localization.^20^, ^21^, ^22^, ^23^ In this study, bioinformatics analysis identified miR‐760 and SOCS3 as the downstream targets of hsa_circ_0007874 (Figure 3A). To confirm that miR‐760 is the hsa_circ_0007874 target, we performed the luciferase reporter assays. The data validated that hsa_circ_0007874 inhibited the luciferase activity in wild‐type cells, while not in mutated cell lines (Figure 3B), advising that hsa_circ_0007874 interacted with miR‐760.

To determine whether SOCS3 was a miR‐760 target, we conducted bioinformatics analyses to detect the potential interaction of miR‐760 with the SOCS3 3'‐UTR (Figure 3C). Luciferase reporter assays showed that miR‐760 inhibited the luciferase activity in wild‐type cells though not in mutated cell lines (Figure 3D). Taken together, the data suggest that silencing hsa_circ_0007874 inhibits the OVA progression by targeting the miR‐760/SOCS3 axis.

### 3.4 Downregulation of SOCS3 or MIR‐760 overexpression restores the migration and proliferation ability of A2780 And SKOV3 cells overexpressing hsa_circ_0007874

**Figure 4:**
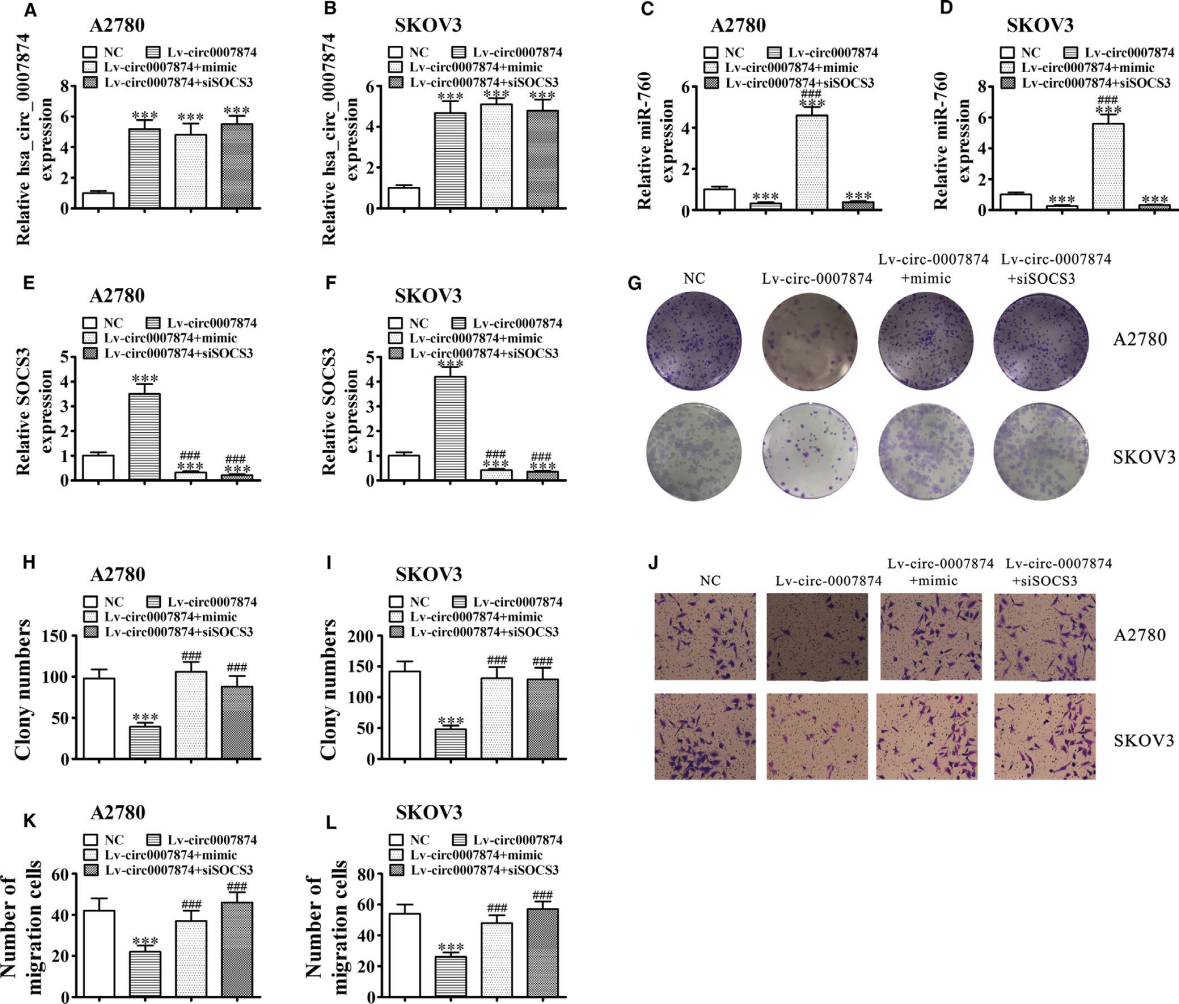
Downregulation of SOCS3 or overexpression of miR‐760 restored the proliferation and migration ability of A2780 and SKOV3 cells overexpressing hsa_circ_0007874. (A‐F) qRT‐PCR detection showing the expression of hsa_circ_0007874 (A and B), miR‐760 (C and D), and SOCS3 (E and F) in A2780 and SKOV3 cells transfected with NC, Lv‐circ0007874, miR‐760 mimics, or siRNA against SOCS3 (siSOCS3) alone or in combination. Data are presented as the mean ± SD. ***P < .001 vs NC. ^###^ *P* < .001 vs Lv‐circ0007874. (G‐I) Colony formation assays. Data are presented as the mean ± SD. ***P < .001 vs NC. ^###^ *P* < .001 vs. Lv‐circ0007874. (J‐L) Cell migration was assessed in A2780 and SKOV3 cells using Transwell assays. Data are presented as the mean ± SD. ^***^ *P* < .001 vs NC. ^###^ *P* < .001 vs Lv‐circ0007874

To validate the interactions between miR‐760, hsa_circ_0007874, and SOCS3, an hsa_circ_0007874 overexpression vector, miR‐760 mimic, and a SOCS3 inhibitor vector were constructed and transfected into OVA cell lines alone or in combination. Rt‐qPCR detection demonstrated that the hsa_circ_0007874 expression significantly increased in A2780 and SKOV3 cells transfected with Lv‐circ0007874. Downregulation of SOCS3 or overexpression of miR‐760 did not restore or affect the hsa_circ_0007874 expression (Figure 4A,B). This suggested that miR‐760 and SOCS3 act downstream of hsa_circ_0007874. Rt‐qPCR detection verified that miR‐760 expression decreased significantly in A2780 and SKOV3 cells transfected with Lv‐circ0007874. Downregulation of SOCS3 did not restore miR‐760, whereas the miR‐760 overexpression significantly increased miR‐760 expression (Figure 4C,D). This suggested that SOCS3 acts downstream of miR‐760. Rt‐qPCR detection confirmed that the SOCS3 expression increased significantly in A2780 and SKOV3 cells transfected with Lv‐circ0007874. Overexpression of miR‐760 or downregulation of SOCS3 significantly decreased the SOCS3 levels (Figure 4E,F), confirming that SOCS3 acts downstream of miR‐760. Clone formation assays (Figure 4G‐I) and Transwell detection (Figure 4J‐L) indicated that downregulation of SOCS3 or miR‐760 overexpression restored migration as well as proliferation ability of SKOV3 and A2780 cells overexpressing hsa_circ_0007874.

### 3.5 Downregulation of SOCS3 restores the migration as well as the proliferation ability of A2780 and SKOV3 cells after the downregulation of MIR‐760 expression

**Figure 5:**
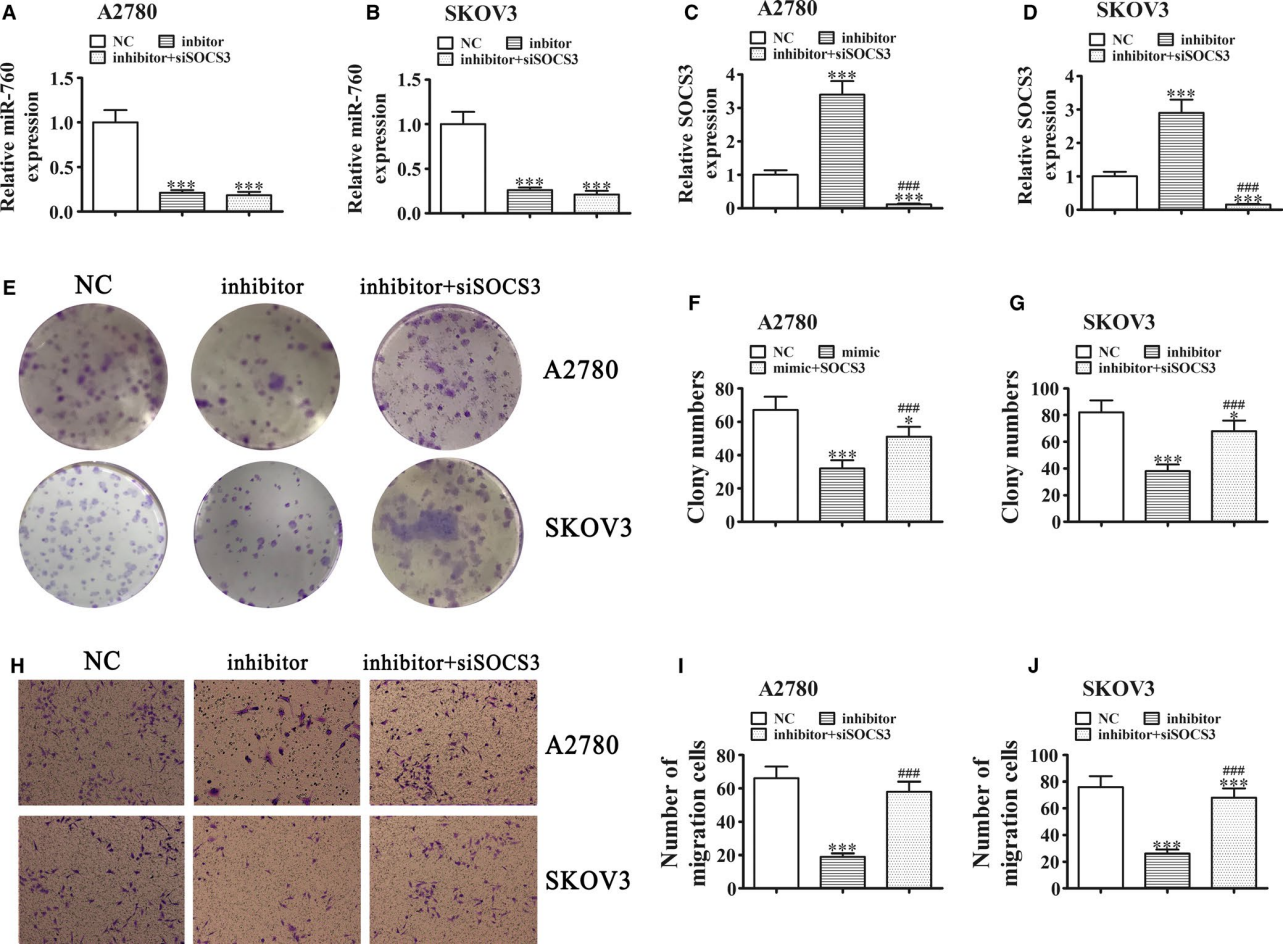
Downregulation of SOCS3 restored the proliferation and migration ability of A2780 and SKOV3 cells transfected with a miR‐760 inhibitor. A‐D, qRT‐PCR detection showing the expression of miR‐760 (A and B) and SOCS3 (C and D) in A2780 and SKOV3 cells transfected with NC, miR‐760 inhibitor, or siSOCS3 alone or in combination. Data are presented as the mean ± SD. ***P < .001 vs NC. ^###^ *P* < .001 vs inhibitor. E‐G), Colony formation assays. Data are presented as the mean ± SD. *P < .05, ***P < .001 vs NC. ^###^ *P* < .001 vs inhibitor. H‐J, Cell migration was assessed in A2780 and SKOV3 cells using Transwell assays. Data are presented as the mean ± SD. ^***^ *P* < .001 vs NC. ^###^ *P* < .001 vs inhibitor

Rt‐qPCR detection showed that A2780 and SKOV3 cell transfected with the miR‐760 inhibitor downregulated the miR‐760 expression, whereas downregulation of SOCS3 did not restore the miR‐760 expression (Figure 5A,B). Rt‐qPCR detection showed that miR‐760 silencing upregulated the SOCS3 expression, whereas downregulation of SOCS3 decreased the SOCS3 expression (Figure 5C,D). Clone formation assays (Figure 5E‐G) and Transwell detection (Figure 5H‐J) showed that downregulation of SOCS3 reversed the miR‐760 silencing effect on inhibiting cell migration and proliferation in A2780 and SKOV3 cells.

### 3.6 Overexpression of hsa_circ_0007874 suppresses OVA growth

**Figure 6:**
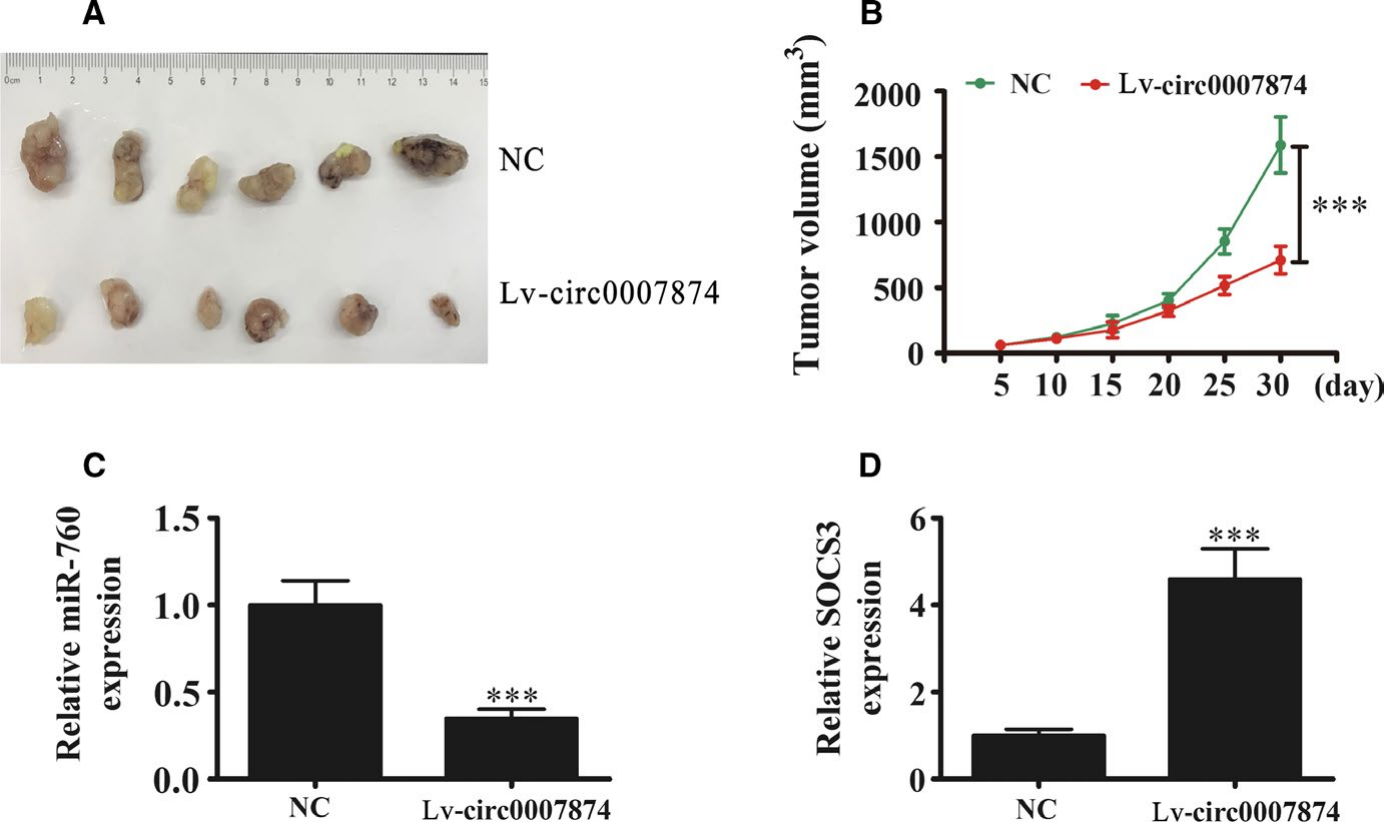
Overexpression of hsa_circ_0007874 suppressed OVA growth. Xenotransplantation studies with the A2780 cell line were performed in BALB/c nude mice. A, Representative images of tumor formation in xenografts of nude mice. B, Tumor volumes were measured every 5 days for 30 days before mouse were murdered. Data are presented as the mean ± SD. ^***^ *P* < .001 vs NC. (C and D) qRT‐PCR detection of miR‐760 expression (C) and SOCS3 (D). Data are presented as the mean ± SD. ^***^ *P* < .001 vs control

Hsa_circ_0007874 overexpressing A2780 cells were used to induce tumor formation in nude mouse xenografts. After 5 days, we measured tumor volume with a Vernier caliper and keep measuring for 30 days before mouse were murdered. The data showed that the hsa_circ_0007874 upregulation suppressed the tumor growth (Figure 6A,B). RT‐qPCR detection confirmed that the miR‐760 expression in tumor tissues significantly decreased in the hsa_circ_0007874 overexpression group (Figure 6C). RT‐qPCR detection also showed that hsa_circ_0007874 upregulation promoted the SOCS3 expression (Figure 6D), suggesting that hsa_circ_0007874 functions in OVA tumorigenesis.

## 4 DISCUSSION

The roles of circRNAs in cancer progression and carcinogenesis have attracted much attention.^24^, ^25^, ^26^ While their function and expression in OVA remain largely elusive. Here, we verified that hsa_circ_0007874 is downregulated in OVA cell lines. hsa_circ_0007874 overexpression significantly decreased the OVA cell proliferation and migration. In vivo studies confirmed that hsa_circ_0007874 overexpression decreased tumor growth, suggesting that hsa_circ_0007874 acts as a tumor suppressor.

Several studies showed that circRNAs act as miRNA sponges.^27^, ^28^ In this study, our analysis identified miR‐760 as a downstream hsa_circ_0007874 target. Luciferase reporter experiments and Rt‐qPCR detection confirmed that miR‐760 can be absorbed by hsa_circ_0007874. Low miR‐760 expression is correlated with higher overall survival in hepatocellular carcinoma patients.^29^ In non‐small cell lung cancer (NSCLC), we showed that radiation can significantly increase the miR‐760 expression in NSCLC cells. miR‐760 knockdown attenuates radiation‐suppressed NSCLC cell proliferation.^30^ In OVA, we validated that the miR‐760 expression is markedly upregulated in OVA tissues and cell lines, and high miR‐760 expression is correlated with poor prognosis and an aggressive phenotype in OVA patients. miR‐760 upregulation promotes, whereas miR‐760 downregulation inhibits the OVA cell proliferation in vitro.^31^ This study also adivsed that miR‐760 downregulation suppressed the OVA cell migration and proliferation. miR‐760 overexpression restored the proliferation and migration ability in cells overexpressing hsa_circ_0007874.

Bioinformatics analysis indicated that miR‐760 interacted with the SOCS3 3'‐UTR. Luciferase reporter experiments and Rt‐qPCR detection confirmed that miR‐760 interacted with the SOCS3 3'‐UTR. miR‐760 downregulation promoted SOCS3 expression. Previous studies validated that SOCS3 expression is downregulated in cancer tissues, including pancreatic cancer, colorectal cancer, lung cancer, bladder cancer, and breast cancer.^32^, ^33^, ^34^, ^35^, ^36^ SOCS3 overexpression has an anti‐proliferative and anti‐metastatic effect in cancer.^37^, ^38^ Previous studies have found that when a cytokine or a growth factor binds to an intracellular receptor as a ligand, the receptor can form a heterodimer and phosphorylate the JAK kinase. Activated JAK can phosphorylate the tyrosine residue of STAT and activated STAT is separated from the receptor complex, forms a dimer, and is translocated from the cytoplasm to the nucleus, where it acts on specific DNA fragments and regulates gene transcription and expression.^39^ SOCS is a negative regulator in the JAK‐STAT signaling pathway and plays an important role in maintaining homeostasis in the cell. As an important tumor suppressor gene, the abnormal expression or function of SOCS3 plays an important role in the occurrence and progression of various tumors, including OVA.^40^

In this study, we showed that downregulation of SOCS3 restored the proliferation as well as migration ability of cells with hsa_circ_0007874 upregulation. Downregulation of SOCS3 also restored the migration and proliferation ability of cells after miR‐760 silencing. Taken together, these data indicate an indispensable tumor‐suppressor role for the hsa_circ_0007874/miR‐760/SOCS3 pathway in OVA.

## 5 CONCLUSIONS

In summary, we verified that the hsa_circ_0007874 expression suppressed oncogenesis by sponging miR‐760, suggesting that hsa_circ_0007874 is a promising OVA prognostic biomarker. In addition, we inferred a novel hsa_circ_0007874/miR‐760/SOCS3 axis that might be used as an effective therapeutic target for the OVA treatment.

## DISCLOSURE

None declared.

## AUTHORS’ CONTRIBUTIONS

LL, PY, PZ, HW, and QC designed the research and drafted the paper with input of all authors. SL and YW conducted the experiments and analyzed the results. LL and HW performed experiments and edited the paper. All authors read and approved the final version.

## References

[1] Siegel R , Naishadham D , Jemal A . Cancer statistics, 2013. CA Cancer J Clin. 2013;63:11‐30. 23335087 10.3322/caac.21166

[2] Luo L , Gao YQ , Sun XF . Circular rna itch suppresses proliferation and promotes apoptosis in human epithelial ovarian cancer cells by sponging mir‐10a‐alpha. Eur Rev Med Pharmacol Sci. 2018;22:8119‐8126. 30556849 10.26355/eurrev_201812_16503

[3] Siegel RL , Miller KD , Jemal A . Cancer statistics, 2017. CA Cancer J Clin. 2017;67:7‐30. 28055103 10.3322/caac.21387

[4] Lv Y , Li H , Li F , Liu P , Zhao X . Long noncoding rna mnx1‐as1 knockdown inhibits cell proliferation and migration in ovarian cancer. Cancer Biother Radiopharm. 2017;32:91‐99. 28414551 10.1089/cbr.2017.2178

[5] Lalwani N , Prasad SR , Vikram R , Shanbhogue AK , Huettner PC , Fasih N . Histologic, molecular, and cytogenetic features of ovarian cancers: Implications for diagnosis and treatment. Radiographics. 2011;31:625‐646. 21571648 10.1148/rg.313105066

[6] Bast RC Jr , Hennessy B , Mills GB . The biology of ovarian cancer: new opportunities for translation. Nat Rev Cancer. 2009;9:415‐428. 19461667 10.1038/nrc2644 PMC2814299

[7] Cannistra SA . Cancer of the ovary. N Engl J Med. 2004;351:2519‐2529. 15590954 10.1056/NEJMra041842

[8] Xue D , Wang H , Chen Y , et al. Circ‐akt3 inhibits clear cell renal cell carcinoma metastasis via altering mir‐296‐3p/e‐cadherin signals. Mol Cancer. 2019;18:151. 31672157 10.1186/s12943-019-1072-5 PMC6824104

[9] Wei J , Wang J , Gao X , Qi F . Identification of differentially expressed circrnas and a novel hsa_circ_0000144 that promote tumor growth in gastric cancer. Cancer Cell Int. 2019;19:268. 31636511 10.1186/s12935-019-0975-y PMC6794874

[10] Shen T , Cheng X , Liu X , et al. Circ_0026344 restrains metastasis of human colorectal cancer cells via mir‐183. Artif Cells Nanomed Biotechnol. 2019;47:4038‐4045. 31608699 10.1080/21691401.2019.1669620

[11] Ma H , Tian T , Liu X , et al. Upregulated circ_0005576 facilitates cervical cancer progression via the mir‐153/kif20a axis. Biomed Pharmacother. 2019;118:109311. 31545253 10.1016/j.biopha.2019.109311

[12] Li XQ , Song JY , Lv W , Zhang D , Wu JZ . Circular circ_0000885 promotes hepatocellular carcinoma proliferation by epigenetically upregulating caprin1. Eur Rev Med Pharmacol Sci. 2019;23:7848‐7854. 31599409 10.26355/eurrev_201909_18994

[13] Chen Y , Ye X , Xia X , Lin X . Circular rna abcb10 correlates with advanced clinicopathological features and unfavorable survival, and promotes cell proliferation while reduces cell apoptosis in epithelial ovarian cancer. Cancer Biomark. 2019;26:151‐161. 31381507 10.3233/CBM-190064 PMC12826436

[14] Wang W , Dong R , Guo Y , et al. Circmto1 inhibits liver fibrosis via regulation of mir‐17‐5p and smad7. J Cell Mol Med. 2019;23:5486‐5496. 31148365 10.1111/jcmm.14432 PMC6653252

[15] Ge Z , Li LF , Wang CY , Wang Y , Ma WL . Circmto1 inhibits cell proliferation and invasion by regulating wnt/beta‐catenin signaling pathway in colorectal cancer. Eur Rev Med Pharmacol Sci. 2018;22:8203‐8209. 30556859 10.26355/eurrev_201812_16513

[16] Li Y , Wan B , Liu L , Zhou L , Zeng Q . Circular rna circmto1 suppresses bladder cancer metastasis by sponging mir‐221 and inhibiting epithelial‐to‐mesenchymal transition. Biochem Biophys Res Commun. 2019;508:991‐996. 30551873 10.1016/j.bbrc.2018.12.046

[17] Sun S , Wang W , Luo X , et al. Circular rna circ‐add3 inhibits hepatocellular carcinoma metastasis through facilitating ezh2 degradation via cdk1‐mediated ubiquitination. Am J Cancer Res. 2019;9:1695‐1707. 31497351 PMC6726993

[18] Zhang B , Chen M , Jiang N , Shi K , Qian R . A regulatory circuit of circ‐mto1/mir‐17/qki‐5 inhibits the proliferation of lung adenocarcinoma. Cancer Biol Ther. 2019;20:1127‐1135. 30975029 10.1080/15384047.2019.1598762 PMC6606010

[19] Liu Y , Dong Y , Zhao L , Su L , Luo J . Circular rnamto1 suppresses breast cancer cell viability and reverses monastrol resistance through regulating the traf4/eg5 axis. Int J Oncol. 2018;53:1752‐1762. 30015883 10.3892/ijo.2018.4485

[20] Chen F , Feng Z , Zhu J , et al. Emerging roles of circrna_nek6 targeting mir‐370‐3p in the proliferation and invasion of thyroid cancer via wnt signaling pathway. Cancer Biol Ther. 2018;19:1139‐1152. 30207869 10.1080/15384047.2018.1480888 PMC6301817

[21] Liu J , Kong F , Lou S , Yang D , Gu L . Global identification of circular rnas in chronic myeloid leukemia reveals hsa_circ_0080145 regulates cell proliferation by sponging mir‐29b. Biochem Biophys Res Commun. 2018;504:660‐665. 30205959 10.1016/j.bbrc.2018.08.154

[22] Chen Q , Zhang J , He Y , Wang Y . Hsa_circ_0061140 knockdown reverses foxm1‐mediated cell growth and metastasis in ovarian cancer through mir‐370 sponge activity. Mol Ther Nucleic Acids. 2018;13:55‐63. 30236833 10.1016/j.omtn.2018.08.010 PMC6143755

[23] Bian L , Zhi X , Ma L , et al. Hsa_circrna_103809 regulated the cell proliferation and migration in colorectal cancer via mir‐532‐3p / foxo4 axis. Biochem Biophys Res Commun. 2018;505:346‐352. 30249393 10.1016/j.bbrc.2018.09.073

[24] Wang S , Zhang Y , Cai Q , et al. Circular rna foxp1 promotes tumor progression and warburg effect in gallbladder cancer by regulating pklr expression. Mol Cancer. 2019;18:145. 31623628 10.1186/s12943-019-1078-z PMC6796492

[25] Wu K , Liao X , Gong Y , et al. Circular rna f‐circsr derived from slc34a2‐ros1 fusion gene promotes cell migration in non‐small cell lung cancer. Mol Cancer. 2019;18:98. 31118036 10.1186/s12943-019-1028-9 PMC6530145

[26] Bach DH , Lee SK , Sood AK . Circular rnas in cancer. Mol Ther Nucleic Acids. 2019;16:118‐129. 30861414 10.1016/j.omtn.2019.02.005 PMC6411617

[27] Chen LL . The biogenesis and emerging roles of circular rnas. Nat Rev Mol Cell Biol. 2016;17:205‐211. 26908011 10.1038/nrm.2015.32

[28] Qu S , Liu Z , Yang X , et al. The emerging functions and roles of circular rnas in cancer. Cancer Lett. 2018;414:301‐309. 29174799 10.1016/j.canlet.2017.11.022

[29] Sun D , Lu J , Hu C , et al. Prognostic role of mir‐760 in hepatocellular carcinoma. Oncol Lett. 2018;16:7239‐7244. 30546462 10.3892/ol.2018.9546 PMC6256363

[30] Zhu L , Xue F , Cui Y , et al. Mir‐155‐5p and mir‐760 mediate radiation therapy suppressed malignancy of non‐small cell lung cancer cells. BioFactors. 2019;45:393‐400. 30901121 10.1002/biof.1500

[31] Liao Y , Deng Y , Liu J , et al. Mir‐760 overexpression promotes proliferation in ovarian cancer by downregulation of phlpp2 expression. Gynecol Oncol. 2016;143:655‐663. 27726922 10.1016/j.ygyno.2016.09.010

[32] Chu Q , Shen D , He L , Wang H , Liu C , Zhang W . Prognostic significance of socs3 and its biological function in colorectal cancer. Gene. 2017;627:114‐122. 28603075 10.1016/j.gene.2017.06.013

[33] Zhang JR , Zhu RH , Han XP . Mir‐410 affects the proliferation and apoptosis of lung cancer a549 cells through regulation of socs3/jak‐stat signaling pathway. Eur Rev Med Pharmacol Sci. 2018;22:5994‐6001. 30280781 10.26355/eurrev_201809_15933

[34] Lin XM , Chen H , Zhan XL . Mir‐203 regulates jak‐stat pathway in affecting pancreatic cancer cells proliferation and apoptosis by targeting socs3. Eur Rev Med Pharmacol Sci. 2019;23:6906‐6913. 31486490 10.26355/eurrev_201908_18730

[35] Li MZ , Lai DH , Zhao HB , Chen Z , Huang QX , Situ J . Socs3 overexpression enhances adm resistance in bladder cancer t24 cells. Eur Rev Med Pharmacol Sci. 2017;21:3005‐3011. 28742207

[36] Xu JZ , Shao CC , Wang XJ , et al. Circtada2as suppress breast cancer progression and metastasis via targeting mir‐203a‐3p/socs3 axis. Cell Death Dis. 2019;10:175. 30787278 10.1038/s41419-019-1382-y PMC6382814

[37] Liu B , Wu S , Ma J , et al. Lncrna gas5 reverses emt and tumor stem cell‐mediated gemcitabine resistance and metastasis by targeting mir‐221/socs3 in pancreatic cancer. Mol Ther Nucleic Acids. 2018;13:472‐482. 30388621 10.1016/j.omtn.2018.09.026 PMC6205337

[38] Ru P , Steele R , Hsueh EC , Ray RB . Anti‐mir‐203 upregulates socs3 expression in breast cancer cells and enhances cisplatin chemosensitivity. Genes Cancer. 2011;2:720‐727. 22207897 10.1177/1947601911425832 PMC3218409

[39] Zhou QY , Peng PL , Xu YH . Mir‐221 affects proliferation and apoptosis of gastric cancer cells through targeting socs3. Eur Rev Med Pharmacol Sci. 2019;23:9427‐9435. 31773681 10.26355/eurrev_201911_19436

[40] Shang AQ , Wu J , Bi F , et al. Relationship between her2 and jak/stat‐socs3 signaling pathway and clinicopathological features and prognosis of ovarian cancer. Cancer Biol Ther. 2017;18:314‐322. 28448787 10.1080/15384047.2017.1310343 PMC5499756

